# What do women living with HIV think about research on children born HIV-free in the UK?

**DOI:** 10.23889/ijpds.v7i3.1823

**Published:** 2022-08-25

**Authors:** Laurette Bukasa, Angelina Namiba, Shema Tariq, Matilda Brown, Estelle Ndungu, Mercy Nangwale, Gillian Letting, Patricia Chirwa, Claire Thorne

**Affiliations:** 1 UCL Great Ormond Street Institute of Child Health; 2 UK4M Network of Mentor Mothers; 3 UCL Centre for Clinical Research in Infection and Sexual Health, Institute for Global Health

## Objectives

We are analysing linked public health surveillance data on pregnancies to women living with HIV in the UK and vital registration data on all their children born HIV-free, an under-researched group. We sought to engage women living with HIV to elicit their feedback on our work and set research priorities.

## Approach

In partnership with 4M Net, a national peer-support network for mothers living with HIV, we co-designed and held two online workshops (one workshop a week over two weeks) in March 2022. We designed the workshops to be highly interactive, using a combination of online tools and created a safe space for open discussion. All participants received a preparatory booklet in advance, comprising questions, activities, and space for reflections during the workshops. We also prompted participants to discuss their research priorities with peers and/or family members if they felt comfortable with this.

## Results

In total, 6 participants attended the two 2-hour long workshops. All participants were from Black ethnic backgrounds, aged 30 years or older and were mothers of children or young adults born HIV-free.

Overall, participants were positive about the programme of research and identified pregnancy, birth, and long-term outcomes in children (especially regarding HIV medication in pregnancy) as being of key importance. They supported the use of linked mental health, hospital, general practice (GP), health visiting and education data to explore pregnancy, health and developmental outcomes among children born HIV-free. Linkage to GP data was identified as a priority by participants, and there was particular interest in addressing knowledge gaps on the mental health of children and young adults born HIV-free.

## Conclusion

Women living with HIV are often involved in research around pregnancy, but rarely in research about their children. By working with trusted community partners, we can engage this often marginalised group of parents who understand the value of linked data research exploring the health and wellbeing of their children born HIV-free.

